# Roots and Root Canals Characterization of Permanent Mandibular Premolars Analyzed Using the Cone Beam and Micro Computed Tomography—A Systematic Review and Metanalysis

**DOI:** 10.3390/jcm12062183

**Published:** 2023-03-11

**Authors:** Mohmed Isaqali Karobari, Ali A. Assiry, Galvin Sim Siang Lin, Hussain Almubarak, Saleh Ali Alqahtani, Robina Tasleem, Mohammed Mustafa, Alexander Maniangat Luke, Krishna Prasad Shetty, Tahir Yusuf Noorani, Giuseppe A. Scardina

**Affiliations:** 1 Conservative Dentistry Unit, School of Dental Sciences, Health Campus, Universiti Sains Malaysia, Kubang Kerian, Kota Bharu 16150, Kelantan, Malaysia; 2 Department of Conservative Dentistry & Endodontics, Saveetha Dental College & Hospitals, Saveetha Institute, Medical and Technical Sciences University, Chennai 600077, Tamil Nadu, India; 3 Preventive Dental Science Department, Faculty of Dentistry, Najran University, Najran 55461, Saudi Arabia; 4 Department of Diagnostic Sciences & Oral Biology, College of Dentistry, King Khalid University, Abha 61471, Saudi Arabia; 5 Restorative Sciences Department (RDS), Operative Dentistry, College of Dentistry, King Khalid University, Abha 62529, Saudi Arabia; 6 Department of Prosthodontics, College of Dentistry, King Khalid University, Abha 62529, Saudi Arabia; 7 Department of Conservative Dental Sciences, College of Dentistry, Prince Sattam Bin Abdulaziz University, Al-Kharj 11942, Saudi Arabia; 8 College of Dentistry, Centre of Medical and Bio-Allied Health Sciences Research, Ajman University, Al-Jruf, Ajman P.O. Box 346, United Arab Emirates; 9 Department of Surgical, Oncological and Stomatological Disciplines, University of Palermo, 90133 Palermo, Italy

**Keywords:** CBCT, dental anatomy, dental pulp, dental diagnostic imaging, endodontics, morphology, micro CT, root, root canal

## Abstract

This current paper aims to review the literature on the root canal configuration (RCC) and root structure of permanent mandibular premolars. To find the published scientific literature on the RCC of mandibular premolars up to July 2022, a systematic search of four electronic databases was performed. The studies were selected, rendering to a predetermined point of reference: “mandibular 2nd premolar”, “root and canal system”, “morphology of root and canal”, “root and canal configuration”, and “morphology”, along with “anatomy” and “mandibular premolars”. Cross-referencing along with screening through the bibliographies of the chosen articles resulted in the identification of further studies. In the current study, we examined 30 different articles, and we chose them based on the quality of research investigations. From 3471 retrieved studies, a total of 15981 mandibular 2nd premolars were observed in 30 studies. The mean JBI score for studies assessing the structure of the root, as well as the root canal of the mandibular 2nd premolar, was 7.78 ± 0.81. We have found a pooled prevalence of 91.82% for Vertucci class I root canal morphology and 78.63% pooled prevalence of single-rooted mandibular second premolar. A total of 8677 mandibular 1st premolars were observed in 22 studies. The mean JBI score for studies assessing the structure and anatomy of the root, as well as root canal of mandibular 1st premolar, was 7. 95 ± 0.85. We have found a pooled prevalence of 74.34% for Vertucci class I root canal morphology and 85.20% pooled prevalence of single-rooted mandibular 1st premolar. Mandibular first and second premolars were mostly single-rooted teeth (89.5–100%). The most frequently seen RCC is a 1-1-2-/2 (type V–Vertucci’s), followed by 1-1-2-/1 (type IV– Vertucci’s; type III–Weine’s), and finally RCC 2-2-2-1 (type IV–Vertucci’s; type III–Weine’s). Presently, the imaging of CBCT is the most used research approach for studying Mn2Ps’ structural characteristics.

## 1. Introduction

Endodontic professionals must know the root canal and admire the morphology and anatomy to provide successful treatment [1]. Endodontic treatment in teeth with necrosed pulp and periapical pathology usually refers to the pulp canal system disinfection, including removing the necrosed pulp and eradicating microbial biofilm from the root canal systems [2]. One of the most reported reasons for root canal treatment failure is frequently attributed to being incapable to find and treat all canals, resulting in persistent bacteriological infection within the missed root canal [3]. Vertucci classified root canal structure and anatomy into type I to type VIII and additional grouping [4]. Most commonly, mandibular premolars have an individual root with an individual canal, yet Cleghorn and his colleagues conducted a comprehensive review of the literature regarding the variation of multiple root canals of the mandibular 1st and 2nd premolar [5,6]. They asserted that the difference in the first mandibular premolars was comparatively higher than the 2nd mandibular premolars, which were mainly discovered in Asia and Africa.

The mandibular 2nd premolar root and morphology of the canal can be extraordinarily difficult and needs careful evaluation before root canal therapy. The event of multiple canals in mandibular 1st premolars is between 11.53% and 46% [7], and the potentiality of a 2nd root canal has been reported to range between 0.4% to 48.1% using cone beam computed tomography (CBCT) [8,9].

Various techniques to analyze the structure of the root canal include tooth clearing technique, tooth sectioning, conventional radiography, CBCT, and micro-computed tomography (micro CT) [10]. However, tooth clearing and sectioning can be useful for in-vitro studies, and they are not typically performed in a clinical setting. They are mainly used for research purposes or as a part of dental education and training. By examining the internal structure of teeth in this way, researchers can gain a better understanding of tooth development, function, and disease processes [7,8,9]. CBCT imaging is an extra-oral radiographic technique, which is the most preferred and consistent method for analyzing the structural and anatomical root canal appearance and root in large populations precisely with low radiation exposure [11]. According to our knowledge, the micro CT was not used by researchers to assess the structure of the mandibular 2nd premolar root canal. The micro CT is a non-destructive, non-invasive, ex vivo high-resolution imaging technique, which, when merged with three-dimensional imaging software, is regarded as the most precise research technique of morphology of root canals and the benchmark in internal and external morphology investigation in the field of endodontics [12].

The literature has shown significant ethnic variations in the mandibular premolar roots and root canals amongst different populations, including the Japanese, Chinese, and Burmese populations [13]. Another study revealed a significant difference concerning gender, age, and ethnicity with variations in mandibular premolars’ root and canal morphology [14]. It is still unclear what effect demographic factors have on the multiple canals found in mandibular premolar teeth. As a result, this study evaluates the disparity inside the root canals of mandibular 1st and 2nd premolars, geographic regions, along with the influence of gender including age on the occurrence of 2nd root with canal among the mandibular premolar using CBCT and micro CT.

## 2. Methodology

### 2.1. Registration and Protocol

Prior to the study, the methodology for this review paper was registered, acknowledged in PROSPERO (CRD42022319884), and developed following the PRISMA (Preferred Recording Items for Meta-Analysis and Systematic Review) Guidelines (http://www.prisma-statement.org, accessed on 12 October 2022).

#### Research Question

Using the micro CT (micro-computed tomography), along with the CBCT (cone beam computed tomography), how similar or how dissimilar is the root, as well as canal morphology, of the human 1st and 2nd premolars?

Population: human mandibular premolars
Intervention: CBCT or Micro CT
Comparison: first premolar and second premolar with normal anatomy
Outcome: anatomy of root, as well as root canal
Study: retrospective radiographic imaging analysis

### 2.2. Search Strategy and Source

Using MESH terms, four electronic databases (ScienceDirect, Google Scholar, Web of Science, and PubMed) were searched to distinguish all existing studies on mandibular 1st and 2nd premolar configurations of roots and canals evaluated using CBCT or micro CT imaging technology (Table 1). A manual search of citations from the extracted articles was also performed. The study authors were contacted via email to gain access to additional information, which could be found in scientific articles, theses, or the grey literature.

### 2.3. The Eligibility Criteria and Study Selection

**Figure 1:**
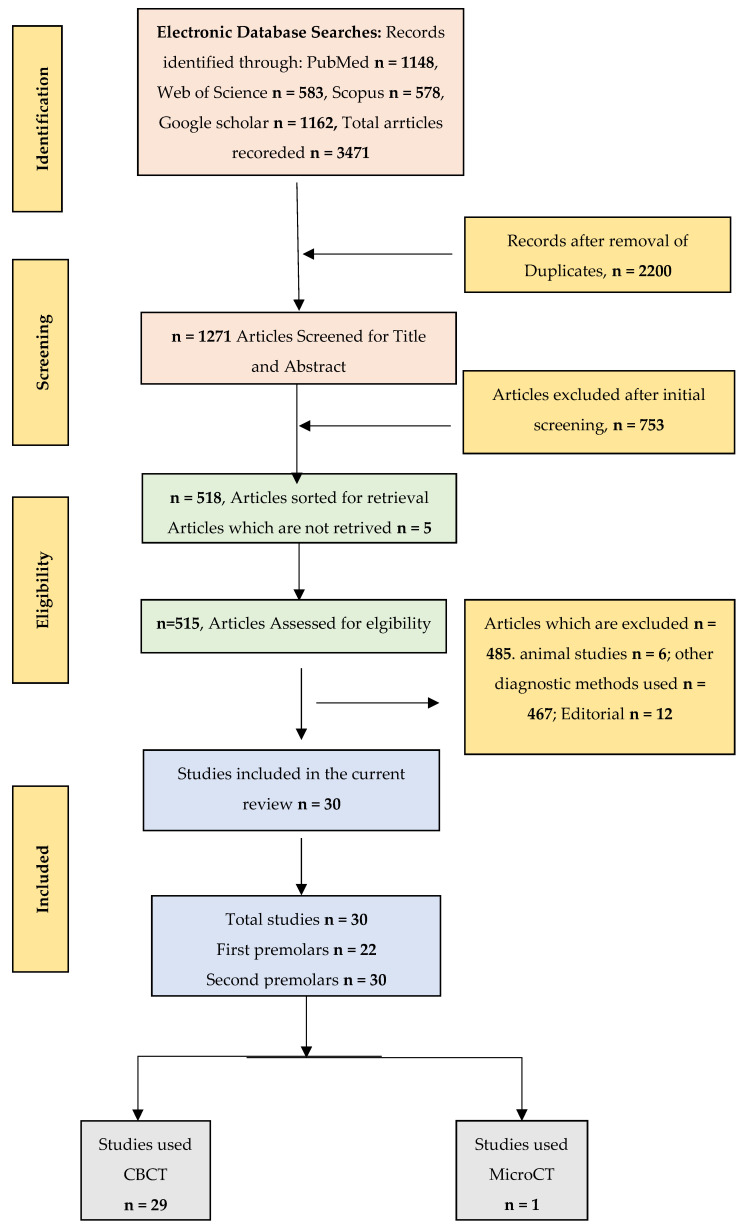
Prisma flowchart.

The articles, which were included, followed the PRISMA guidelines 2020 (Figure 1) for the selection of final eligible studies. All the studies featuring roots and their canal morphology of mandibular premolars, following the standard classification system using CBCT or MicroCT with the JBI critical appraisal tool score of more than 50%, were included for the study and non-clinical studies, as well as studies including root canal-treated teeth, and editorials and case reports were excluded.

### 2.4. Data Sources and Extraction

The data collection was performed for the data published until May 2022, using data which were extracted on request without concealing bibliographic record data, titles, or authors. No language restrictions were imposed. Studies that lacked a complete understanding of case reports and only defined the teeth as premolars or mandibular premolars were omitted.

Duplicate or recurring articles were rejected after correlating the findings from the four selected databases (ScienceDirect, PubMed, Google Scholar, and Web of Science). Two independent authors (MIK and TYN) evaluated the abstracts and titles of the obtained articles, and if they were deemed pertinent, the respective full-text articles were taken into consideration. The year of the article, the duration of the study, baseline participant information, and root canal configuration data were all recorded when available.

Tables were created from the equivalent results, including pertinent facts. Two independent reviewers (MIK and TYN) assessed the abstracts of the obtained articles and decided whether or not to include them in the systematic review. As a result, articles that did not fit the criteria for inclusion were rejected, and those that did were restored in pdf format. Using the Vertucci classifications, the occurrence of a configuration root canal, the total amount of roots and teeth, and the source of the samples examined by Briseo Marroqun et al. and Weine et al. were summarized in a table format.

The inner structure of the root is broken down into the coronal, middle, and apical third directions by Marroqun B et al. using a four-digit approach. The first three digits, which are separated by a dash (-), reflect the coronal margins of the middle, coronal, and apical thirds. The 4th digit, which is distinct from the remaining three numbers by a slash (/), speaks for the number of apical portals of exit. Senior faculty took decisions in case of any dispute in the inclusion of articles.

### 2.5. Assessment of Scientific Merit

The JBI (Joanna Briggs Institute) Critical Appraisal method critically assessed systematic reviews of preponderance studies, considering the scientific validity of the selected studies. In Table 2, The JBI queries were graded as “applicable/not applicable” or “yes/no” for each applicable study. However, the final mark for this paper only considered “yes” answers. Two evaluators worked separately to complete this evaluation. The interrater reliability between the two evaluators was estimated using the Cohen kappa value. A score =/> 0.80 was considered to be a good agreement score. Before a final agreement was established between the two evaluators, the major evaluation divergences were reviewed. The only thing taken into consideration for each study’s final score was constant agreement.

### 2.6. Statistical Analysis

Based on the distribution given in the collaborative investigations, the preponderance of extra roots, including canals in mandibular 1st and 2nd premolars, was determined. The Comprehensive Meta-Analysis software’s random-effects model was used to process all of the data. Odds ratios (OR) and the fraction of forest plots along a 95% confidence interval (CI) were used to represent the final results. To assess the degree of heterogeneity between the studies, tau 2 (assessment of in-between variance of study) was used. To quantify the statistical heterogeneity of the suggested outcomes, I^2^ statistics were used. Depending on the I^2^ value (%), the variability was described as “low” (25%), “moderate” (50%), or “high”. *p* < 0.05 was used as the analytical significance level.

## 3. Results

### 3.1. Study Excerpt

The results of our search included 1148 research papers that made use of MeSH keywords, 583 research papers from Web of Science, 578 research papers from Scopus, and 1162 research papers from Google Scholar. A total of 1271 studies were recognized for further review after the removal of the duplicate articles, which totaled 2200. After reading the titles of every item, another 753 were disqualified from consideration. In addition, a total of 485 further papers were eliminated from consideration after reading the articles’ abstracts and entire texts, which were then assessed for further selection. The information was taken from the 30 studies that were completely qualified to participate in the analysis. The selection criteria depicted in Figure 1 are consistent with the PRISMA guidelines. For the present research, these 30 articles were analyzed in terms of the standard of the research studies.

### 3.2. Study Features

The studies are subdivided further according to their authors, the year, the reference number, the demography, the number of teeth that were analyzed, the methods that were used during the study, the configuration, and the number of the root canal and roots frequency (%). The attributes of the studies were depicted in Table 3.

#### 3.2.1. First Premolars

**Figure 2:**
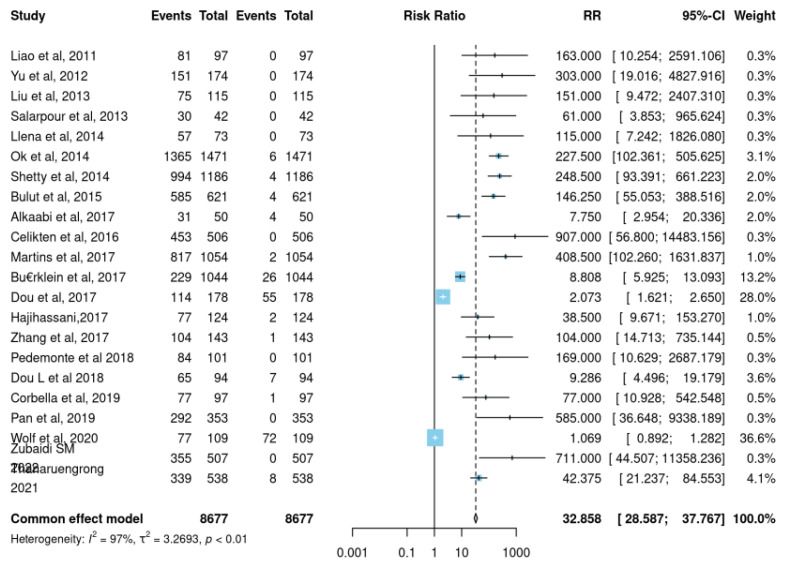
Forest plot for the prevalence of more than one root canal against a single root canal in the first premolar [6,7,9,15,16,17,18,19,21,24,27,30,33,37,38].

A total of 8677 mandibular 1st premolars were observed in 22 studies. The mean JBI score as shown in Table 4 for studies evaluating the root morphology alongside its canal in mandibular 1st premolar was 7. 95 ± 0.85. Table 3 shows how we have found a pooled prevalence of 74.34% for Vertucci class I root canal morphology and 85.20% pooled prevalence of single-rooted mandibular 1st premolar. The pooled prevalence of other morphological classifications was mentioned in Table 5. We performed a common effect model for pooled prevalence and observed significantly high heterogeneity (I^2^ = 97%; *p* = 0.001) amongst the studies (Figure 2).

#### 3.2.2. Second Premolars

**Figure 3:**
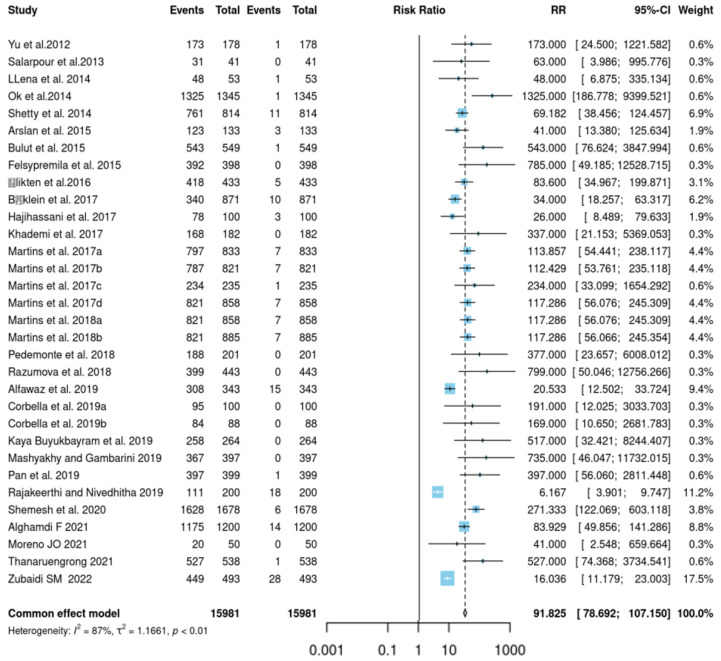
Forest plot for the prevalence of more than one root canal against a single root canal in the second premolar [2,6,7,9,13,15,16,17,18,19,20,21,22,23,24,25,26,27,28,29,30,31,32,33,34,35,36,37,38,39].

A total of 15,981 mandibular 2nd premolars were observed in 30 studies. The mean JBI score for studies evaluating the root and canal morphology of mandibular 2nd premolar was 7.78 ± 0.81. Table 3 shows that we have found a pooled prevalence of 91.82% for Vertucci class I root canal morphology and 78.63% pooled prevalence of single-rooted mandibular 2nd premolar. The pooled prevalence of other morphological classifications was mentioned in Table 2. We performed a common effect model for pooled prevalence and observed significantly high heterogeneity (I^2^ = 87%; *p* = 0.001) among the studies (Figure 3).

### 3.3. Second Root Canal and Root Prevalence Concerning Gender

**Figure 4:**
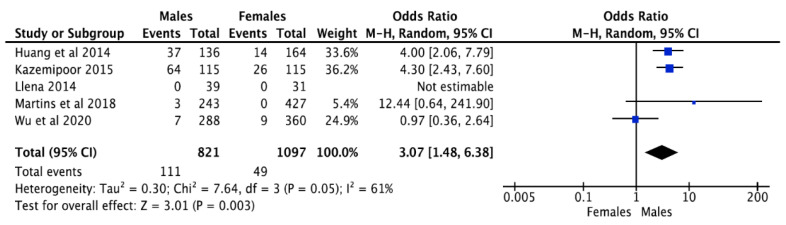
Prevalence of more than one root canal in mandibular first premolar according to gender [17,40,41,42,43].

The second root prevalence concerning gender was directed in five studies [15,16,17,18,19], while that of the root canal was mentioned in six of the included studies [15,16,17,18,19,20]. For both outcomes, males were dominant over females with regards to the root numbers, along with the canals for both the 1st and 2nd premolars. The second root overall prevalence in males was 69.3% (111 out of 160), while, in females, it was 30.6% (49 out of 160). Five studies (accounting for 40 roots in total) [21,22,23,24,25] mentioned only the overall prevalence of the second root, but they did not comment on the relative prevalence according to the second root concerning gender. The overall pooled analysis had a Z score of 3.01, with a *p*-value of <0.003. The odds ratio for 2nd root prevalence in males and females was 3.07 (1.48 ± 6.38), indicating an increased frequency of occurrence of the second root in males compared to the females. The I^2^ value was 61%, representing a lower heterogeneity among the selected studies (Figure 4).

**Figure 5:**
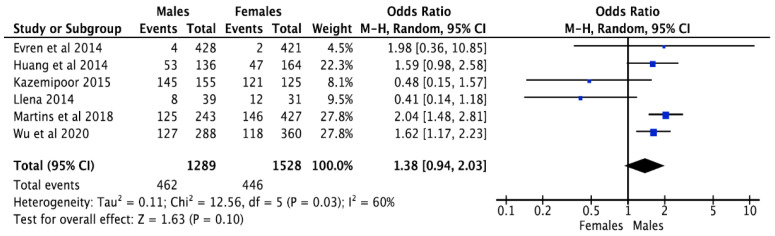
Prevalence of more than one root canal in mandibular second premolar, according to gender [17,40,41,42,43].

**Figure 6:**
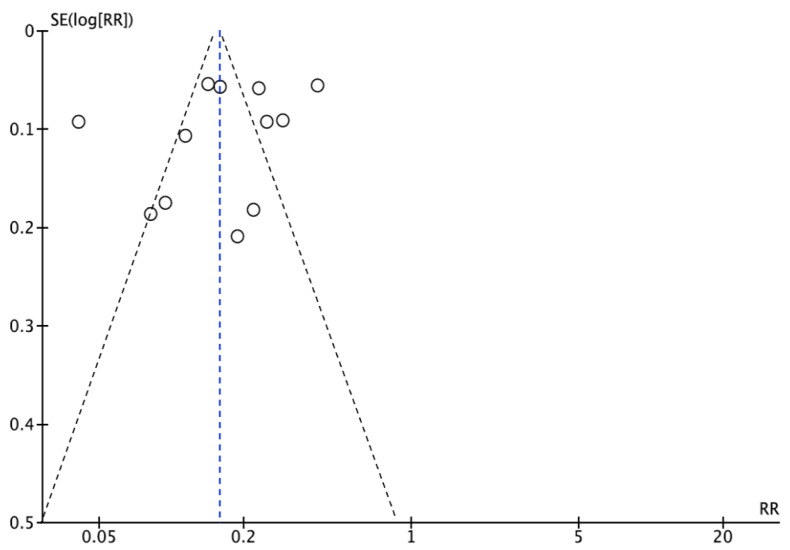
Funnel plot for publication bias.

In terms of second root canal prevalence, 53.9% (546 out of 1013) of the males had the incidence of a 2nd root canal, while 46.2% (468 out of 1013) of the females showed the occurrence of a 2nd root canal. Of the six studies conducted [21,22,23,24,25,26], there was no mention of gender distribution in the prevalence of the 2nd root canal. The collaborative analysis about the incidence of the 2nd root canal, concerning gender, had a Z score of 1.63 with a *p*-value of 0.10, and the odds ratio was 1.38 (0.94 ± 2.03). Because of the 95% CI for odds ratio, the 2nd root canal prevalence includes measurements above and below 1, and the 2nd root canal prevalence was comparable in both males and females. The I^2^ value for the 2nd root canal prevalence was 60%, indicating a low to moderate diversity among the included studies (Figure 5). The bias of publication, if any, was addressed by the funnel plot (Figure 6) that represents adequate measurements on both sides of the graph.

According to the geographic area, the second root canal and the root prevalence are calculated for people from Asia and Europe. As per the six studies [15,16,17,21,23,24] conducted, the occurrence of a second root is more prevalent in the Asian continent. These studies represented people from China, Iran, and the province of Taiwan. The prevalence of the second root was 4.23% (181/4277) of the mandibular premolars. The overall pooled analysis for the above-mentioned regional studies had a Z score of 8.20 (*p* < 0.00001) along with an odds ratio that was calculated to be at 0.00 (0.00 ± 0.00), which indicated a significantly low prevalence for the second root among Asians. Similarly, the prevalence of the second root from the people belonging to Europe has been calculated. Four studies [18,19,22,25] mentioned the 2nd root of mandibular premolars from people belonging to Portugal, Spain, and France. The second root was found in 0.4% of the mandibular premolars (19/4730). The Z score for the pooled analysis was calculated at 4.91 (*p* < 0.00001), and the estimated odds ratio was 0.00 (0.00 ± 0.00), which was significantly lower in the European population.

**Figure 7:**
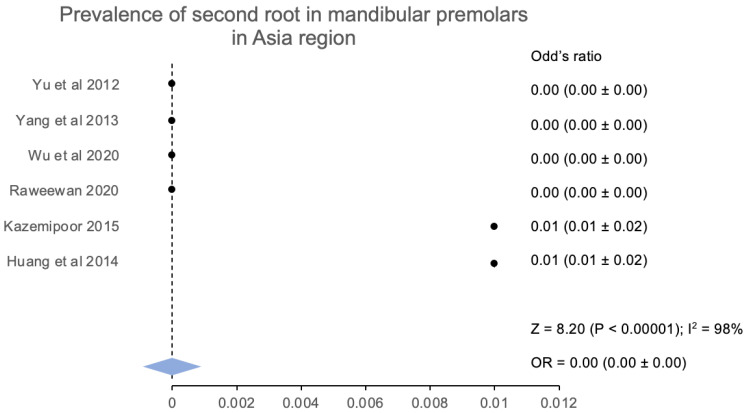
Prevalence of more than one root in first mandibular premolars in the Asia region [15,42,43,44].

**Figure 8:**
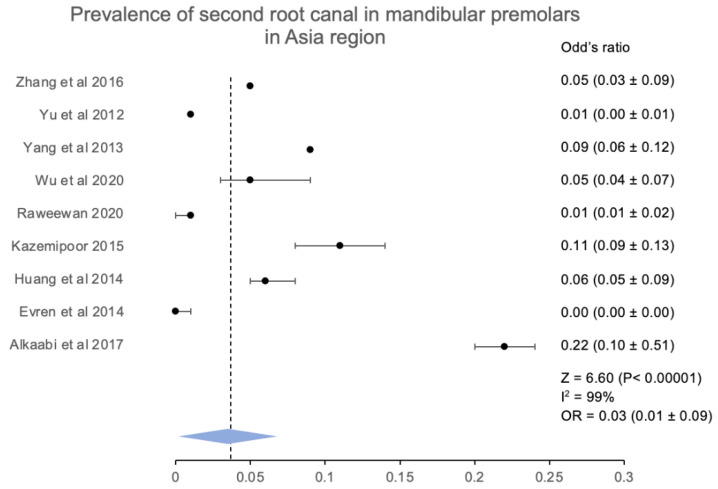
Prevalence of more than one root in the second mandibular premolar Asia region [15,42,43,44].

According to the occurrence of second root canals in Asian and European populations, nine studies from Asia [15,16,17,20,21,23,24,27,28] and four studies from Europe [18,19,22,25] confirmed the occurrence of second root canals. The occurrence was stated from countries such as China, Iran, Turkey, Thailand, Dubai, and the province of Taiwan, which turned out to be 13.32% (971/7286) of the mandibular premolars in the Asian population. The overall collaborative analysis had a Z score of 6.60 (*p* < 0.00001) along with an odds ratio that was calculated to be at 0.03 (0.01 ± 0.09), which indicated a significantly low prevalence for the second root canal among Asians. (Figure 7 and Figure 8).

**Figure 9:**
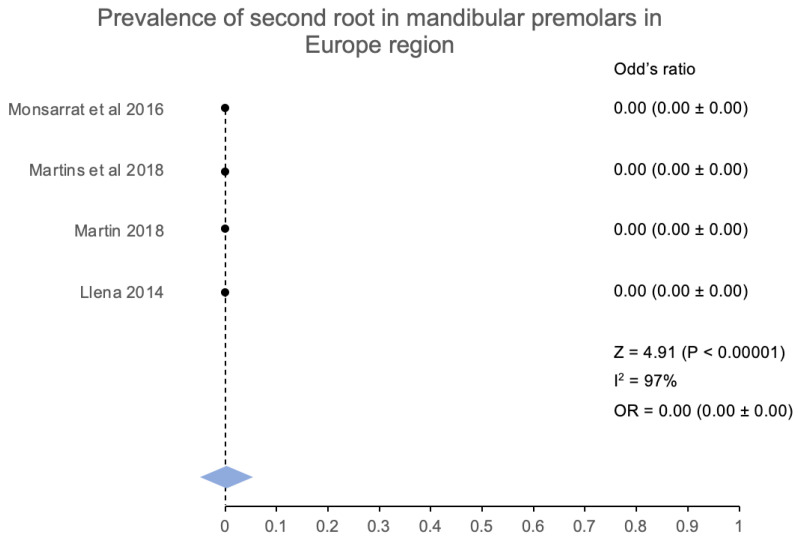
Prevalence of more than one root canal in mandibular first premolar in the Europe region [17,25,26,45].

**Figure 10:**
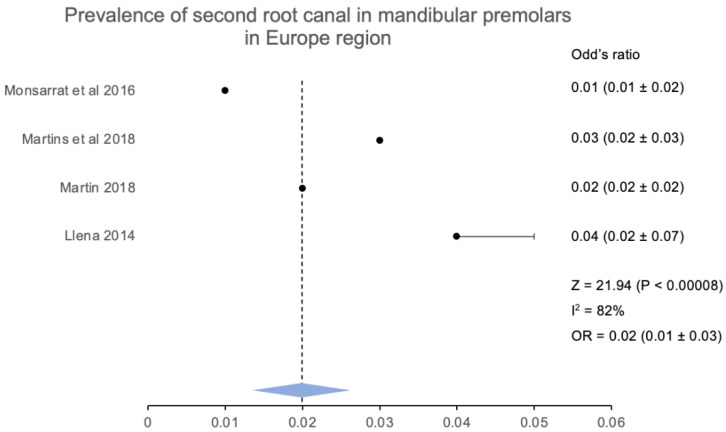
Prevalence of more than one root canal in mandibular second premolar in the Europe region [17,25,26,45].

The people of Europe have also been assessed similarly (Figure 9), which indicated an increase in the occurrence of the second root canal. Furthermore, four studies that were conducted later confirmed the preponderance of 2nd root canals in people of Spain, France, and Portugal. The occurrence of a second root canal in this subgroup of the population was 12.87 (623/4840), while the overall Z score for the pooled analysis was calculated at 21.94 (*p* < 0.00001), and the odds ratio was estimated to be 0.02 (0.01 ± 0.03), which was again significantly lower in the European population (Figure 10).

**Figure 11:**
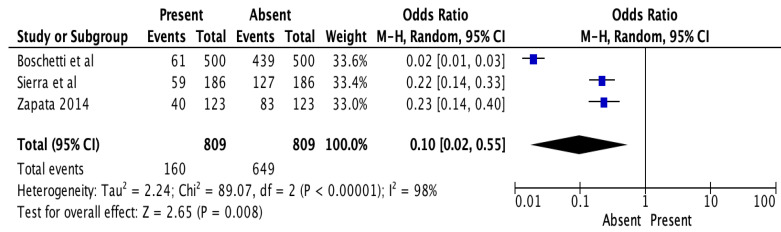
Prevalence of more than one root in mandibular premolars in Latin America [46,47,48].

In addition, two studies [29,30] mentioned the occurrence of a 2nd root canal in the Brazilian population, while one study commented on the demographics of the population from Chile [31]. The prevalence of the 2nd root from these South American countries was 1.29% (4/309) of the mandibular teeth, while the prevalence for the 2nd root canal was 19.77% (160/809). The overall Z score for the prevalence of the 2nd canal was 2.65 (*p* < 0.008) with an odds ratio of 0.10 (0.02 ± 0.55), which indicates a lower prevalence for the 2nd root canal in the Latin American population (Figure 11). Due to the small number of studies, the odds ratio for the occurrence of the 2nd root was not calculated.

## 4. Discussion

This study provides a detailed analysis of the internal anatomy of mandibular premolar teeth using advanced imaging techniques. The study highlights the variability of root and canal morphology in these teeth and identifies several anatomical variations that may not be detectable with traditional radiography. The use of CBCT and micro CT in this study allowed for a comprehensive understanding of the internal structure of these teeth, which is essential for clinicians in the diagnosis and treatment of dental conditions. The study’s findings can help improve the accuracy and success of endodontic procedures, such as root canal therapy, by enabling clinicians to identify and treat anatomical variations that may have been previously missed.

The internal morphology and anatomy of a root canal can be efficiently assessed and investigated by micro CT. This three-dimensional imaging technique is reproducible and non-destructive, with high-resolution accuracy. Despite being used widely, CBCT does not provide the same high-resolution accuracy of the root canal morphology as micro CT [25,49]. More than half of the published studies that were looked at for this report were carried out with the help of the CBCT imaging technique.

Yu et al. initially introduced the imaging method being discussed in the context of studying Mn2Ps [15]. CBCT, on the other hand, is a useful technique for studying large sample sizes, as well as for conducting in vivo studies due to its speed and ease of use. Furthermore, CBCT has the added advantage of being free from reports of intra-observer discrepancies [9,25].

In this review study, the sample size for Mn2Ps ranged from 40 to 1678 teeth [35,50], with the majority of the included studies utilizing samples of more than 100 teeth. The findings of the reviewed studies indicated that a large proportion of Mn2Ps was single-rooted, with prevalence rates ranging from 89.5% to 100% [2,6,7,15,16,19,20,23,25,27,28,29,30,31,32,33,34,35,50,51,52,53,54,55,56,57]. The presence of two roots in Mn2Ps was observed in 0.1% to 8% of cases [2,6,7,20,27,35,54,55,56], with Pawar and Singh [55] reporting the highest prevalence and Martins et al. reporting the lowest [23,24,26]. Mn2Ps with three roots were sporadically observed in 0.1% to 3.5% of cases [34,51]. Notably, Rajakeerthi and Nivedhitha found that three-rooted Mn2Ps and RCCs have a comparatively higher prevalence in the Indian population [34].

Vertucci’s RCC system [58] was found to be the most frequently used method in the studies reviewed, with five studies employing this approach [14,28,46,59,60]. Marroqun et al. [52] RCC 1-1-1/1 (Weine’s Type I and Vertucci’s method) was the most commonly reported (55.3%–99.6%) in all of the studies evaluated, except for Bürklein et al.’s study [7], in which a RCC 1-1-2/2 (type V–Vertucci’s) was most commonly observed, while the prevalence of 1-1-1/1 (Weine’s type I and Vertucci’s) RCCs was low (39%). The RCC 1-1-2/2 (Type V–Vertucci’s) was the second most commonly observed RCC in Mn2Ps in around 50% of the evaluated research, with a frequency range of 0.5% to 57.1% being reported [4,6,7,8,15,16,17,19,21,22,23,24,25,27,34,50,54,58]. Salarpour et al. [16] discovered a comparatively high incidence (22%) of 1-1-2/2 (type V–Vertucci’s) RCCs through the use of CBCT technology. Bulut et al. [6] found that, in a study of 549 Mn2Ps using CBCT technology, 98.5% of the sample had 1-1-1/1 (Weine’s and Vertucci’s type I) RCCs, while only 0.5% had a 1-1-2/2 (type V–Vertucci’s) RCC.

Among the reviewed studies, the second most common RCC was the 2-2-2/2 (type IV in Vertucci’s classification and type III in Weine’s), with a prevalence ranging from 0.6% to 18% in almost one-third of the studies evaluated [14,18,28,29,30,42,43,44,49,59,60,61,62,63]. Sert and Bayirli [63] reported that this RCC had the highest prevalence (18%) in the male group. The 2-2-1/1 RCC (type II in Weine’s and Vertucci’s classifications) had a low frequency (0.1% to 10.8%) [2,4,5,6,9,15,17,18,19,20,21,22,23,24,25,32,34,35,51,52,53,60,63], but Singh and Pawar found the highest repetition rate of 30% in Mn2Ps [60]. Among all the reviewed studies (Table 1), other RCCs, such as the 2-1-2/2, 1-2-1/1, 1-2-1/2, and 1-1-3/3 (types VIII, VII, VI, and III in Vertucci’s classification) were reported occasionally.

According to Vertucci’s [50] classification, four of the examined investigations [14,59,60,61,62] were published. Regarding one to two root canals in Mn2Ps, Green et al. [14], Pineda and Kuttler [59], and Miyoshi et al. [62] have all reported findings. Depending on the RCCs from Weine et al. [64], Vertucci [58], and Briseo Marroqun et al. [61], the appropriate results have been grouped (Table 1). Based on the studies we reviewed, a root canal can be classified as Weine’s and Vertucci’s type I if it has a Briseo Marroqu’s 1-1-1/1 RCC [14,59,62]. On the other hand, double root canals are equivalent to Weine’s type III, Vertucci’s type IV, and Briseo Marroqun’s 2-2-2/2 RCC.

The studies we reviewed reported that Mn2Ps (mandibular second premolars) mostly had a single root canal, ranging from 92% to 98.8%, while 1.2% to 8% had two root canals [14,59,62]. However, Dowson and Zillich [60] identified a rare case of an additional root canal in Mn2Ps, which was classified as a 2-2-1/1 RCC type II of Briseo Marroqun and Vertucci and Weine’s [14,59,60,62]. This RCC needs to be treated with caution, as it was not previously distinguished from the other root canal types in Mn2Ps. The frequency of 2-2-2/2 RCC (type IV–Vertucci’s and type III–Weine’s) in Mn2Ps may be affected by this finding. Gender differences were observed in seven of the twelve papers included in the meta-analysis [2,21,22,25,26,27,32,34,35,40,63]. Women were found to be more likely to have a single root canal in studies that used CBCT imaging, except for the investigation by Sert and Bayirli [54]. The incidence of single-rooted Mn2Ps was high in both genders (89.5–100%), while the incidence of two-rooted Mn2Ps was extremely rare (0–7%). However, Alfawaz et al. [2] and Rajakeerthi, as well as Nivedhitha [34], reported a low incidence of three-rooted Mn2Ps (1.2% and 3.5%, respectively).

Various approaches have been used to compare root canal morphology. In a study by Khademi et al. [22], the results of CBCT scans of 182 mandibular first and second premolars were compared, and a high rate of agreement (87%) between the two methods was reported. The highest level of agreement was observed in 2-2-2/2 RCCs (type IV–Vertucci’s and type III–Weine’s), while the lowest level of agreement was found in 1-1-2/2 RCCs (type V–Vertucci’s). The researchers noted that CBCT was highly accurate in identifying C-shaped root canals, but less accurate in identifying lateral canals. Pedemonte et al. [27] reported that a 1-1-1/1 RCC (type I of Vertucci’s and Weine’s) was consistently found in mandibular premolars in both Chilean (95.0%) and Belgian (92.1%) populations. Furthermore, Martins et al. [26], based on a comparison of CBCT data from the western European and Chinese populations, found that the incidence of 1-1-1/1 RCCs (type I of Vertucci’s and Weine’s) was slightly higher in Chinese mandibular premolars (99.6%) than in Western European mandibular premolars (95.7%).

Most Mn2Ps have a single root, but caution is necessary when analyzing the internal morphology of root canals in different studies, since some authors do not always mention the number of roots being examined. Briseo Marroqun et al. [61] highlight this issue with their 2-2-2/2 RCC classification, which describes the root canal morphology of a single root, unlike Vertucci’s [58] and Weine et al.’s [64] classifications, which treat the tooth and roots as a single entity.

The current investigation found that RCC 1-1-1/1 was detected in 70.6% of the Ma1Ps analysed, which is consistent with results in 65% of cases, but it is not statistically relevant to those described by other authors [2,4,17,18,21,23,27,40,51,54]. The conclusions of other study groups have generated a great deal of debate, as some studies have reported an incidence of this configuration, ranging from 21.9% to 69.0% in their populations [7,9,14,19,51,56], while others have reported an incidence of between 76.0% and 94.2% [8,13,24,26,30,31,32,33,53,58,59]. It is worth noting that Pedemonte et al. [27] found significantly different frequencies of this arrangement in two groups: Belgian (58.3%) and Chilean (56.9%).

In our investigation, we discovered that the three most common root canal configurations (RCCs) out of the total cases (16.4%) were similar to the type V configuration described by Vertucci [20]. These were the 1-1-2/2 (7.3%), 1-2-2/2 (7.3%), and 1-1-1/2 (1.8%) RCCs. These findings were consistent with those reported by other researchers on Egyptians (16.4%), Jordanians (16.8%), and Chinese (17.5%, CBCT imaging) populations, such as Alhadainy, Awawdeh and Al-Qudah, and Zhang et al., respectively [51]. However, the reported RCC frequencies by different authors have varied widely, ranging from 0% to 28.8% (see Table 2). The discrepancies in the findings may be due to differences in study design, sample size, ethnicity, gender, analysis, and RCC techniques, as suggested by some studies [19,60,61,65].

Several studies, including previous micro CT research [6,14,51,56], have revealed inconsistencies in the three-dimensional root canal system of Ma1Ps. Consequently, the success of endodontic treatment relies on two critical factors: firstly, a comprehensive understanding of the anatomy and structure of the root canal system to be treated, enabling the dentist to make an informed decision on the appropriate cleaning and shaping techniques, including the instruments to be used; and secondly, the expertise of the dentist performing the procedure.

## 5. Conclusions

Mandibular first and second premolars were mostly single-rooted teeth (89.5–100%). The most frequently seen RCC is the 1-1-2-/2 (type V–Vertucci’s), followed by a 1-1-2-/1 (type IV–Vertucci’s and type III–Weine’s), and finally a RCC 2-2-2-1 (type IV–Vertucci’s and type III–Weine’s). Presently, CBCT imaging is the most used research procedure for studying Mn2Ps’ structural characteristics. Even though the vast bulk of Mn2Ps have a single root and a single channel (1–1–1/1), endodontic treatments should be planned and executed accordingly, even during the most challenging situations with complicated RCCs.

## Figures and Tables

**Table 1:** Sources and searched strategies information.

Database	Searched Strategies	Results
PubMed	“configuration of root and canal” OR “morphology root and canal” OR “root and canal system” AND “mandibular premolars AND “morphology” OR “anatomy”AND “Cone-Beam Computed Tomography” OR “MICRO CT”	1148
Science Direct	“mandibular premolars” AND “morphology of root and canal” AND “anatomy” OR “morphology” AND “CBCT” OR “MICRO CT”	578
Web of Science	“CBCT” OR “Micro CT” AND “Premolar” AND “morphology”	583
Google Scholar	“mandibular Premolar “,”CBCT”,”Micro CT”,”Root canal”	1162
	Total	3471

**Table 2:** JBI (Joanna Briggs Institute) Critical Appraisal kit of prevalence study questions for systematic reviews.

Was the frame of sample appropriate in addressing the selected population? Was recruiting process for the study samples proper? Was the number of samples being adequate? Was the study setting along with subjects described clearly? Was the analysis of data performed with enough detail of the sample identification? Were accurate procedures applied for confirming the study condition? Was the study condition analyzed acceptably and reliably for each participant? Was the rate of response satisfactory, and if not, was the less response rate handled appropriately? Was there a suitable statistical analysis?

**Table 3:** The characteristics of the included studies.

Author	Country	Sample Size	RCC-Frequency (%)									Roots (*n*; %)			
	RCC		Ve (1984)	I	II	III	IV	V	VI	VII	VIII		1	2	3
			We (1969)	I	II		III								
			Br (2015)	1–1–1/1	2–2–1/1	1–2–1/1	2–2–2/2	1–1–2/2	2–1–2/2	1–2–1/2	1–1–3/3				
Yu et al., 2012 [15]	CHN	178	CBCT	97.2	0.55	0	0	1.7	0	0	0	0.55	100	0	0
Salarpour et al., 2013 [16]	IRN	41	CBCT	75.6	–	–	–	22	–	–	–	2.4	100	0	0
LLena et al., 2014 [17]	ESP	53	CBCT	90.6	1.8	–	–	7.5	–	–	–	–	100	0	0
Ok et al., 2014 [18]	TUR	1345	CBCT	98.5	0.1	0.07	0.6	0.5	0	0	0.2	–	–	–	–
Shetty et al., 2014 [19]	IND	814	CBCT	93.5	1.4	0.2	0	3.9	0	0	0.1	0.7	100	0	0
Arslan et al., 2015 [20]	TUR	133	CBCT	92.4	2.2	0.7	0	1.5	0	0	0	2.9	96.2	3.8	0
Bulut et al., 2015 [6]	TUR	549	CBCT	98.9	0.2	0.4	0	0.5	0	0	0	0	98.9	1.1	0
Felsypremila et al., 2015 [13]	IND	398	CBCT	98.4	0	0	0	0.8	0	0	0	0.8	–	–	–
Çelikten et al., 2016 [9]	TUR	433	CBCT	96.6	1.1	1.1	–	1.1	–	–	–	–	–	–	–
Bürklein et al., 2017 [7]	GER	871	CBCT	39	1.1	0.1	1.4	57.1	0.5	0.3	0.3	0	98.6	1.3	0.1
Hajihassani et al., 2017 [21]	IRN	57	CBCT	80.7	1.8	7	0	8.8	1.8	0	0	0	100	0	0
			F												
		43	M	74.7	4.7	16.3	0	4.7	0	0	0	0	100	0	0
Khademi et al., 2017 [22]	IRN	182 (Ma2P & Ma1P)	CBCT	92.3	0	2.2	2.2	3.3	0	0	0	0	–	–	–
Martins et al., 2017 [23]	PRT	833	CBCT	95.7	0.8	1.3	0.5	1.4	–	–	–	–	99.9	0.1	0
Martins et al., 2017 [23]	PRT	821	CBCT	95.8	0.8	1.2	0.5	1.3	0	0	0	0.3	99.9	0.1	0
Martins et al., 2017 [24]	CHN	235	CBCT	99.6	0.4	0	0	0	0	0	0	0	100	0	0
	PRT	858		95.7	0.8	1.3	0.5	1.4	0	0	0	0.3	99.9	0.1	0
Martins et al., 2018 [25]	PRT	331	CBCT	94.3	0.6	2.1	0.6	1.5	0	0	0	0.9	99.7	0.3	0
			M												
		527	F	96.6	0.9	0.8	0.4	1.3	0	0	0	0	100	0	0
Martins et al., 2018 [26]	PRT (age ≤ 20)	13	CBCT	69.2	0	7.7	0	7.7	0	0	0	15.4	–	–	–
		251	21–40	98.8	0	0.4	0	0.8	0	0	0	0	–	–	–
		395	41–60	96.2	1.3	1.3	0.2	0.8	0	0	0	0.2	–	–	–
		199	≥ 61	92.5	1	2	1.5	3	0	0	0	0	–	–	–
Pedemonte et al., 2018 [27]	BEL	101	CBCT	92.1	–	3	–	5	–	–	–	1	98	2	0
Comp	CHL	100		95	–	2	–	2	–	–	–	0	99	1	0
											–				
Razumova et al., 2018 [28]	RUS	443	CBCT	90.1	.	.	9.9	–	–	–	–	–	99.8	0.2	0
Alfawaz et al., 2019 [2]	SAU	172	CBCT	90.1	3.5	0	1.7	1.2	0	0	3.5	0	95.3	3.5	1.2
			F												
		171	M	90.1	5.3	0.6	3.5	0.6	0	0	0	0	95.9	4.1	0
Corbella et al., 2019 [29]	ITA	100	CBCT	95	0	1	4	0	0	0	0	0	97	3	0
Corbella et al., 2019 [30]	ITA	88	CBCT	95.5	0	0	4.5	0	0	0	0	0	96.6	3.4	0
Kaya Buyukbayram et al., 2019 [31]	TUR	264	CBCT	97.7	0	1.1	0	0.38	0	0	0	0.8	100	0	0
Mashyakhy and Gambarini 2019 [32]	SAU	188	CBCT	94.7	0	2.7	0	1.6	0	0	0	1.1	100	0	0
			M												
		191	F	99	0	0.5	0	0	0	0	0	0.5	100	0	0
Pan et al., 2019 [33]	MYS	399	CBCT	99.5	0.3	–	0.3	–	–	–	–	–	100	0	0
Rajakeerthi and Nivedhitha 2019 [34]	IND	200	CBCT	55.3	8.8	6.1	4.4	15.8	3.5	1.8	4.4	0	89.5	7	3.5
			M												
			F	57	7	8.1	5.8	9.3	2.3	5.8	4.7	0	90.7	5.8	3.5
Shemesh et al., 2020 [35]	ISR	1678 (M 831/F 847)	CBCT	96.4	0.6	1.7	0.1	0.6	0	0	0.1	0.5	99.5	0.5	–
			M												
			F	97.6	0.1	1.1		0.5	0	0	0.3	0.2	99.8	0.2	–
Alghamdi [36] F 2021	SaudiArabia	1200 600 M, 600 F	CBCTM	97.5	1.33	0.17	0.67	0.17	0.17				98.83	1.17	
			F	98.33	1	0	0.5	0.17	0				97.83	2.17	
Zubaidi SM [37] 2022	SaudiArabia	493	CBCT	(91.10)	(5.7)	(0.2)	(2.8)	0 (0)	1 (0.2)	0 (0)	0 (0)		99.2	0.8	
Thanaruengrong [38] 2021	Thailand	538	CBCT	98	0.2	0.4		1.5					537 (99.8)	1 (0.2)	–
Moreno [39] JO 2021	Brazil	50	Micro CT	40		4		24		4		28			

**Table 4:** JBI score for studies included in the analysis.

Study	Country	Total JBI Score
Yu et al., 2012 [15]	CHN	7
Salarpour et al., 2013 [16]	IRI	9
Llena et al., 2014 [17]	ESP	7
Ok et al., 2014 [18]	TUR	8
Shetty et al., 2014 [19]	IND	8
Bulut et al., 2015 [6]	TUR	9
Celikten et al., 2016 [9]	TUR	8
Martins et al., 2017 [23]	PRT	7
Bu€rklein et al., 2017 [7]	GER	7
Hajihassani et al., 2017 [21]	IRI	7
Pedemonte et al. 2018 [27]	BEL	9
Corbella et al., 2019 [30]	ITA	7
Pan et al., 2019 [33]	MAS	9
Thanaruengrong et al., 2021 [38]	Thailand	8
Zubaidi SM et al., 2022 [37]	Saudi Arabia	9

**Table 5:** The pooled prevalence of other morphological classification in first premolar.

RCC-Frequency Numerical									Roots Numerical			
Ve (1984)	I	II	III	IV	V	VI	VII	VIII		1	2	3
We (1969)	I	II		III								
Br (2015)	1–1–1/1	2–2–1/1	1–2–1/1	2–2–2/2	1–1–2/2	2–1–2/2	1–2–1/2	1–1–3/3				
Pooled prevalence%	74.34	2.2	2.5	3.75	15.45	0.44	0.13	0.1	0.95	85.20	11.20	2.50

## Data Availability

The data used in the current study will be made available upon reasonable request.
